# Phenotypical characterisation of a putative ω-amino acid transaminase in the yeast *Scheffersomyces stipitis*

**DOI:** 10.1007/s00203-018-1608-x

**Published:** 2018-12-06

**Authors:** Tomas Linder

**Affiliations:** Department of Molecular Sciences, Swedish University of Agricultural Sciences, Box 7015, 750 07 Uppsala, Sweden

**Keywords:** Antifungal, Metabolism, Phenotype, Reverse genetics, Yeast

## Abstract

Phylogenetic analysis of class III transaminases in the budding yeasts *Lachancea kluyveri, Saccharomyces cerevisiae* and *Scheffersomyces stipitis* identified a hitherto uncharacterised *Sch. stipitis* transaminase encoded by the *PICST_54153* gene, which clustered with previously described γ-amino butyric acid (GABA) and β-alanine transaminases. Deletion of the *PICST_54153* gene in *Sch. stipitis* resulted in a complete loss in the utilisation of β-alanine and β-ureidopropionic acid as nitrogen sources, while growth on 1,3-diaminopropane displayed a significant lag phase compared to the wild-type control. It was therefore concluded that the *Sch. stipitis PICST_54153* gene likely encodes a β-alanine transaminase. However, minor growth defects when 1,4-diaminobutane or 1,5-diaminopentane was provided as the nitrogen source suggested that the Picst_54153 transaminase may also participate in the catabolism of other diamine-derived ω-amino acids. Unexpectedly, the *∆picst_54153* deletion mutant failed to grow on solid minimal medium in the presence of 5 mM β-alanine even if a preferred nitrogen source was provided.

## Introduction

**Fig. 1:**
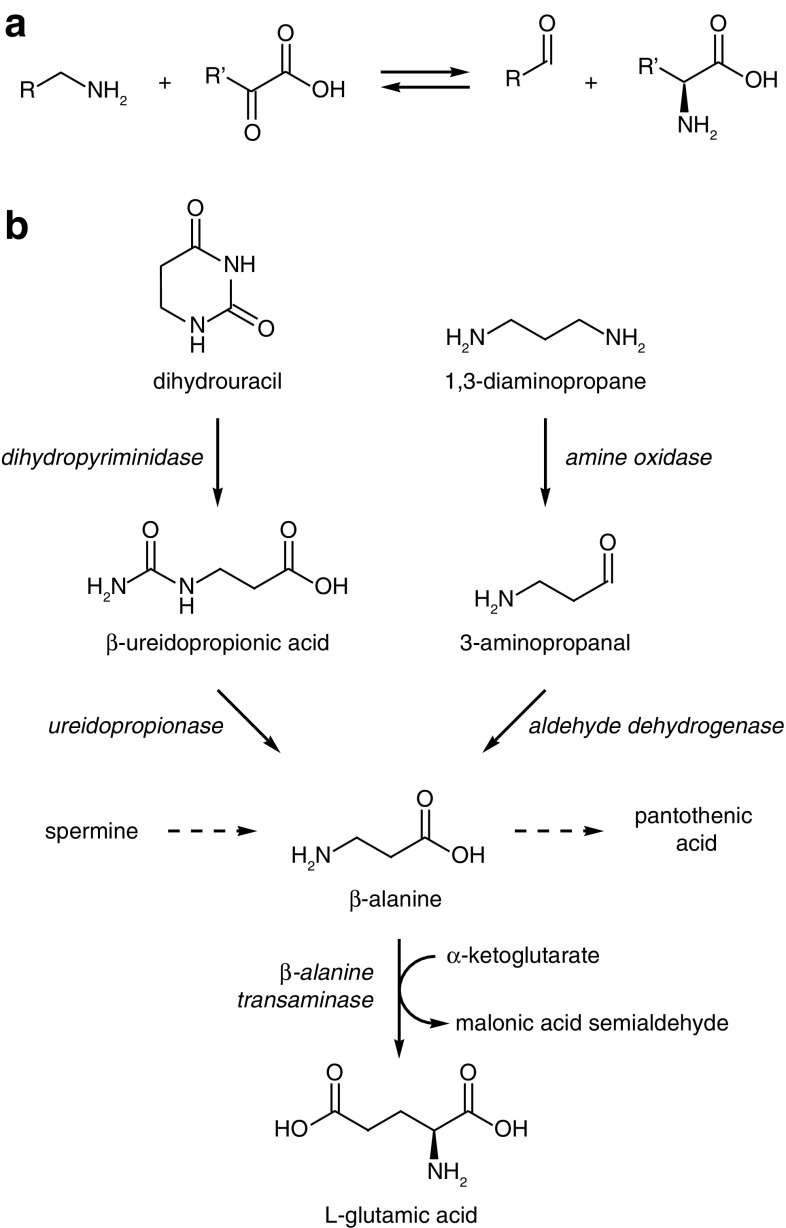
**a** The reaction catalysed by class III transaminases such as Arg8, Car2 and Uga1 in *Sac. cerevisiae*. **b** The proposed catabolic pathways in budding yeasts where β-alanine is an intermediate

Transaminases are pyridoxal 5′-phosphate-dependent enzymes that catalyse the exchange of an amino group and a carbonyl group between two substrates. In yeast, transaminases play a central role in the synthesis and breakdown of predominantly amino acids, but also other biomolecules. Transaminases belonging to class III (EC 2.6.1; Pfam accession PF00202) that have been previously described in the baker’s yeast *Saccharomyces cerevisiae* include acetylornithine transaminase (encoded by the *ARG8* gene; Heimberg et al. 1990), 7,8-diaminononanoate transaminase (encoded by the *BIO3* gene; Phalip et al. 1999), l-ornithine transaminase (encoded by the *CAR2* gene; Degols et al. 1987) and γ-aminobutyric acid (GABA) transaminase (encoded by the *UGA1* gene; André and Jauniaux 1990). In the case of Arg8, Car2 and Uga1 proteins, all catalyse the interconversion of a primary amine and an α-ketoacid (α-ketoglutarate) to an aldehyde and an l-α-amino acid (Fig. 1a).

The amino acid β-alanine is an intermediate in a number of metabolic pathways in yeast, which includes the biosynthesis of pantothenic acid from spermine as well as the breakdown of dihydrouracil (but not uracil itself) and 1,3-diaminopropane (Fig. 1b). *Sac. cerevisiae* does produce β-alanine during pantothenic acid biosynthesis (White et al. 2003), but cannot utilise β-alanine as a nitrogen source (Di Carlo et al. 1952) because it lacks the requisite transaminase. The related yeast *Lachancea kluyveri* has been shown to possess a β-alanine transaminase (encoded by the *PYD4* gene), which enables its utilisation of β-alanine as nitrogen source (Andersen et al. 2007; Schnackerz et al. 2008). The *L. kluyveri* Pyd4 enzyme converts β-alanine and α-ketoglutarate to l-glutamic acid and malonic acid semialdehyde (Schnackerz et al. 2008). l-glutamic acid in turn is a central amino group donor compound for many transamination reactions in intracellular nitrogen metabolism. The *PYD4* gene also appears to be partially redundant with *UGA1* gene for the utilisation of GABA as a nitrogen source in *L. kluyveri* (Andersen et al. 2007). At the time of writing, no further studies have been conducted on the role of class III transaminases in the utilisation of β-alanine as a nitrogen sources by yeasts.

The present study sought to investigate the diversity of class III transaminases in the yeast *Scheffersomyces stipitis* and attempted to identify individual transaminase-encoding genes that play a role in the utilisation of ω-amino acids (H_2_N–[CH_2_]_*n*_–COOH) and linear diamines (H_2_N-[CH_2_]_*n*_-NH_2_) as nitrogen sources.

## Materials and methods

### Sequence alignment and phylogenetic analysis

Class III transaminase homologues in the genomes of *Sac. cerevisiae* and *Sch. stipitis* were identified through a PSI-BLAST search of the GenBank reference sequence (refseq) protein database using the *L. kluveri* Pyd4 protein sequence (GenBank protein accession ABF58893) as query and applying an expected (*E*) value inclusion threshold of 10^−6^. The protein complement of the *L. kluveri* genome was investigated for class III transaminase homologues using the iGenolevures GRYC BLASTP server (http://igenolevures.org/) with an *E* value threshold of 10^−6^. Protein sequences were aligned in MAFFT (Katoh et al. 2005; http://mafft.cbrc.jp/alignment/server/index.html) using the G-INS-i alignment setting. Selection of sequence positions suitable for phylogenetic analysis was carried out in GBlocks (Castresana 2000; http://molevol.ibmb.csic.es/Gblocks_server/), which was configured to allow for smaller final blocks and less strict flanking positions. The resulting amino acid positions were then used to construct a neighbour-joining (NJ) tree using the online MAFFT phylogeny server (Katoh et al. 2017; https://mafft.cbrc.jp/alignment/server/phylogeny.html) using a JTT amino acid substitution model. Branch support was tested using 1000 bootstrap replicates. The consensus trees were visualised in Phylo.io (Robinson et al. 2016).

### *Sch. stipitis* integration constructs

**Fig. 2:**
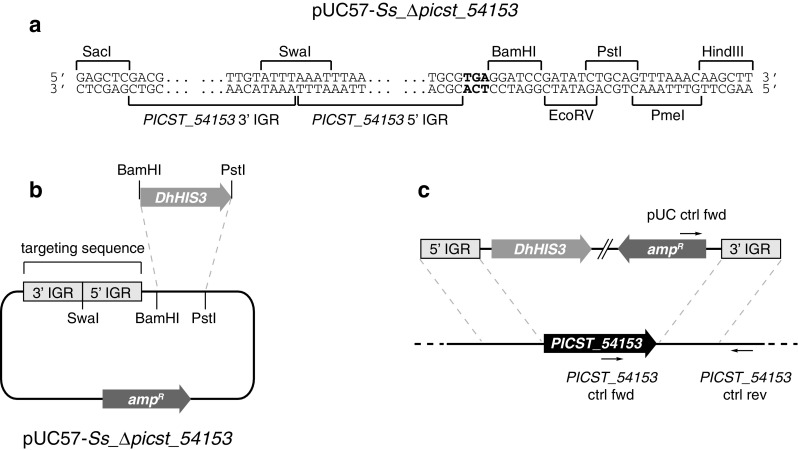
Design of the *Sch. stipitis PICST_54153* gene replacement construct. **a** A targeting cassette consisting of the 5′ and 3′ intergenic regions (IGR) for *PICST_54153* were synthesised in reverse order and inserted into the pUC57 plasmid. An in-frame stop codon (highlighted in bold font) was added to the targeting cassette as the 5′ flanking sequence included a 69-bp portion of the 5′ coding sequence of the *PICST_54153* gene. A short polylinker was incorporated at the 3′ end of the targeting construct to facilitate insertion of a selection marker. **b** Insertion of the BamHI/PstI-cut *DhHIS3* selection marker into the polylinker adjacent to the targeting cassette. **c** The resulting pUC57-*Ss_Δpicst_54153-DhHIS3* plasmid was linearised by digestion with SwaI, which enabled homologous recombination with *PICST_54153* flanking regions

A targeting cassette for deletion of the hypothetical gene *PICST_54153* was synthesised de novo by GenScript (New Jersey, USA) and inserted into SacI/HindIII-cut pUC57 to make plasmid pUC57-*Ss_∆picst_54153* (Fig. 2a). The *PICST_54153* targeting cassette consisted of two tandem 500-bp sequence elements identical to the intergenic region (IGRs) immediately downstream (3′ IGR; GenBank accession NC_009042, residues 375,698–376,197) and immediately upstream (5′ IGR; GenBank accession NC_009042, residues 373,812–374,311) of the *PICST_54153* coding sequence. The 5′ IGR sequence element also included the first 69 bp of the *PICST_54153* coding sequence and an in-frame stop codon was therefore added. The 3′ and 5′ IGR sequences were separated by a SwaI recognition site. The full-length *HIS3* gene was amplified from *Debaryomyces hansenii* CBS 767 genomic DNA with primers DhHIS3 fwd (5′ GCG CGC GGA TCC TTT CAC CAG ATG GGA TCT AAT 3′) and DhHIS3 rev (5′ GCG CGC CTG CAG GCG CGC CAG TCG TAA TGT TTA TAG AAG A 3′), digested with BamHI and PstI and inserted into BamHI/PstI-cut pUC57-*Ss_∆picst_54153* to make plasmid pUC57-*Ss_∆picst_54153*-*DhHIS3* (Fig. 2b). Prior to transformation, pUC57-*Ss_∆picst_54153*-*DhHIS3* was digested with SwaI to produce a linear integration construct, which was then purified into sterile water using the QIAquick PCR purification kit (Qiagen).

### Yeast transformation

The *Sch. stipitis* parent strain SF1 (*his3-1 trp5-10 YKU80::ScTRP5*), which lacks a functional non-homologous end-joining DNA repair pathway (Maassen et al. 2008), was kindly provided by Prof Ulrich Klinner (Aachen University, Germany). The transformation methodology has been described previously (Linder 2014). Correct chromosomal integration and the deletion of the *PICST_54153* gene was confirmed by PCR analysis of purified genomic DNA from the transformed strain (Fig. 2c). Correct integration at the *PICST_54153* locus was assayed using primers pUC ctrl fwd (5′ TCG CCA TTC AGG CTG CGC AAC TGT 3′) and *PICST_54153* 3′ ctrl rev (5′ CCA CCA CAC CAT TCC TGC TGT 3′), which produce no product in the SF1 parent strain and the TLSS001 reference strain (Linder 2014), but a 953-bp amplification product in the *∆picst_54153* strain. Successful deletion of the *PICST_54153* gene was assayed using primers *PICST_54153* ctrl fwd (5′ GTG GCT GTG GCG ATA TGG CTA 3′) and *PICST_54153* 3′ ctrl rev, which produce a 880-bp amplification product in the SF1 parent strain and the TLSS001 reference strain but no product in the *∆picst_54153* strain. The verified *∆picst_54153* strain was assigned strain number TLSS011.

### Yeast growth assays

Sodium l-glutamate, glycine, GABA and β-ureidopropionic acid were purchased from Sigma-Aldrich (Schnelldorf, Germany). β-alanine, δ-aminovaleric acid and the dihydrochloride salts of 1,3-diaminopropane, 1,4-diaminobutane (putrescine) and 1,5-diaminopentane (cadaverine) were purchased from TCI Europe N.V. (Zwijndrecht, Belgium). All growth assays employed a reduced sulphur/nitrogen-limited glucose medium (RSNLD), which only contains trace amounts of nitrogen in the form of vitamins (Linder 2018). Nitrogen utilisation assays in liquid RSNLD medium with individual nitrogen sources were performed as described previously (Linder 2018). For growth assays on solid RSNLD media, 20 g/l agarose was included. Yeast strains were pre-cultured overnight in 3 ml minimal glucose medium (MMD), which consists of 6.7 g/l Difco yeast nitrogen base without amino acids (Becton, Dickinson and Company) and 20 g/l glucose. Pre-cultures were then washed once in sterile water and then diluted to OD_600_ 0.1 in sterile water. 2 µl of this cell suspension was deposited onto RSNLD agarose plates containing the indicated concentrations of primary nitrogen source and the indicated concentration of β-alanine. Plates were photographed after 6 days incubation at 30 °C.

## Results

The *PYD4* gene in *L. kluyveri* encodes the sole β-alanine transaminase identified in budding yeasts thus far. Both *Sac. cerevisiae* and *L. kluyveri* belong to the family Saccharomycetaceae within the budding yeasts (sub-phylum Saccharomycotina). An effort was therefore made to select another yeast species outside the Saccharomycetaceae, which would then be investigated for the degree of functional conservation of class III transaminases in the catabolism of β-alanine as well as other ω-amino acids in this species and thereby provide further perspective on this process among budding yeasts. The yeast *Sch. stipitis* belongs to the family Debaryomycetaceae and is therefore only distantly related to *Sac. cerevisiae* and *L. kluyveri*. This family is distinguished by its use of an alternative genetic code by which the codon CUG is translated as serine rather than leucine (Santos and Tuite 1995). *Sch. stipitis* also possesses a more versatile metabolsism than either *Sac. cerevisiae* or *L. kluyveri*, which includes its ability to ferment the pentose sugar xylose. Based on the taxonomic distance of *Sch. stipitis* from the Saccharomycetaceae as well as its previously known metabolic properties, *Sch. stipitis* was therefore selected for the present study.

**Fig. 3:**
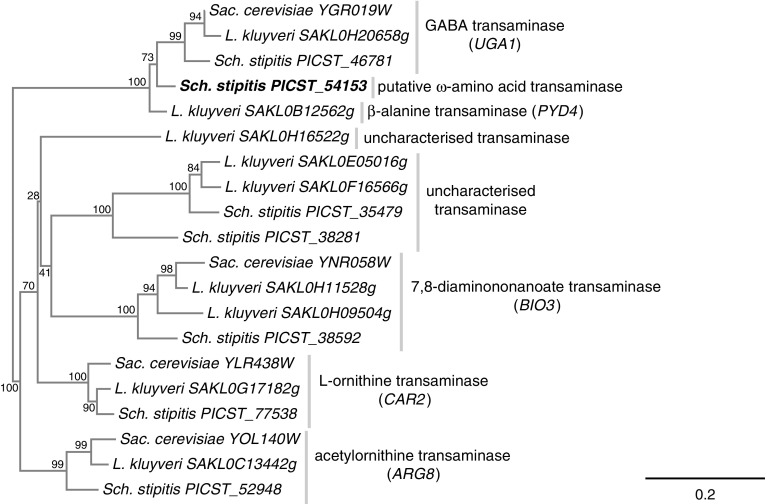
Phylogenetic analysis of class III transaminases in *Sac. cerevisiae, L. kluveri* and *Sch. stipitis* based on 193 aligned amino acid positions of the corresponding gene products. Well-supported monophyletic clades containing a *Sac. cerevisiae* orthologue are labelled with the common *Sac. cerevisiae* gene name and function. Branch labels indicate the frequency of retained nodes among 1000 bootstrap replicates. The *Sch. stipitis* transaminase encoded by the *PICST_54153* gene, which was the subject of the present study, is indicated in bold font

The full complement of class III transaminases in all three species was established through protein sequence similarity searches. The retrieved protein sequences were then aligned, subjected to phylogenetic analysis using the NJ method and visualised in the form of a tree diagram (Fig. 3). The previously described *Sac. cerevisiae* class III transaminases (encoded by the *ARG8, BIO3, CAR2* and *UGA1* genes, respectively) all formed well-supported monophyletic clades with their corresponding *L. kluyveri* and *Sch. stipitis* orthologues. In most cases, *L. kluyveri* and *Sch. stipitis* appeared to possess a single corresponding orthologue to the *Sac. cerevisiae* gene with the exception of the biotin biosynthetic gene *BIO3* where the *L. kluyveri BIO3* orthologue appeared to have undergone a gene duplication event. The genomes of *L. kluyveri* and *Sch. stipitis* also contained two genes each encoding putative transaminases of unknown function, which formed a separate and well-supported monophyletic clade. In addition, the *L. kluyveri* genome also contained a unique putative transaminase (systematic gene name *SAKL0H16522g*), which clustered independently within the dataset. The product of the *L. kluyveri PYD4* gene (GenBank protein accession ABF58893) formed a basal branch to the well-supported cluster including orthologues of the *Sac. cerevisiae* GABA transaminase encoded by the *UGA1* gene. This cluster also contained the protein sequence of a gene encoding a hitherto uncharacterised putative *Sch. stipitis* transaminase (systematic gene name *PICST_54153*; GenBank protein accession XP_001382305). The corresponding protein sequences of the *L. kluyveri PYD4* and *Sch. stipitis PICST_54153* genes did not form a supported clade, which could suggest independent duplications of the ancestral *UGA1* gene in both species.

Catabolism of β-alanine has not previously been studied in *Sch. stipitis* or closely related species. The location of the *Sch. stipitis PICST_54153* gene product within the phylogenetic tree of yeast class III transaminases (Fig. 3) suggested that the protein would act as an ω-amino acid transaminase, although the substrate specificity could not be predicted from phylogentic data alone. The *PICST_54153* gene was therefore deleted in *Sch. stipitis* to elucidate its function (Fig. 2a–c). The resulting deletion mutant displayed no discernable growth defects on rich or minimal medium (data not shown). To determine whether the *PICST_54153* gene plays a role in ω-amino acid catabolism, it was cultivated in chemically defined minimal medium lacking metabolically available nitrogen (Linder 2018), which was then supplemented with individual nitrogen sources to a final concentration of 10 mM total nitrogen. The reference strain TLSS001 (Linder 2014), which contains an intact *PICST_54153* gene, was used as a control. The tested nitrogen sources included four ω-amino acids (glycine, β-alanine, GABA, δ-aminovaleric acid), three linear diamines (1,3-diaminopropane, 1,4-diaminobutane, 1,5-diaminopentane) and the ureido compound β-ureidopropionic acid. The sodium salt of l-glutamic acid was used as a control substrate.

**Fig. 4:**
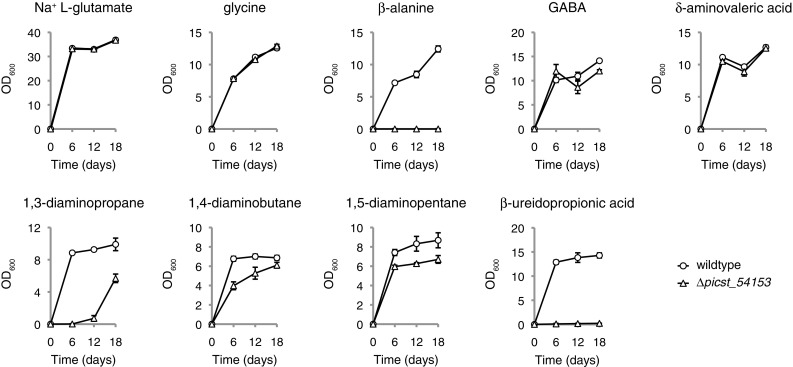
The requirement for the *PICST_54153* gene for the utilisation of ω-amino acids, diamines and β-ureidopropionic acid as sole nitrogen sources. *Sch. stipitis* strains TLSS001 (wildtype control) and TLSS011 (*∆picst_54153*) were cultured in 3 ml RSNLD medium (Linder 2018) supplemented with 10 mM total nitrogen of the indicated nitrogen source (initial OD_600_ 0.005). Samples were incubated in a shaker set at 30 °C, 200 r.p.m., and OD_600_ was measured after 6, 12 and 18 days. Growth assays were performed in triplicate with error bars indicating one standard deviation

The *∆picst_54153* strain was indistinguishable from the reference strain when sodium l-glutamate or glycine was provided as the sole nitrogen source (Fig. 4). Growth on the ω-amino acids GABA and δ-aminovaleric acid were essentially identical between the mutant and the reference strain, while the mutant failed to show any detectable growth when β-alanine was provided as the sole nitrogen source. Growth on the diamines 1,4-diaminobutane and 1,5-diaminopentane was slightly impaired in the *∆picst_54153* mutant compared to the reference, while a severe growth lag was observed in the mutant when 1,3-diaminopropane was provided as the sole nitrogen source. The *∆picst_54153* mutant failed to grow when β-ureidopropionic acid was provided as the sole nitrogen source. These results indicate that the *PICST_54153* gene encodes a transaminase mainly specific for β-alanine, although it appears to play a minor role in transamination of GABA and δ-aminovaleric acid derived intracellularly from deaminated 1,4-diaminobutane and 1,5-diaminopentane, respectively. Its role in the transamination of external GABA and δ-aminovaleric acid appears to be negligible.

The growth defects observed when the *∆picst_54153* mutant was cultivated on either 1,3-diaminopropane or β-ureidopropionic acid as sole nitrogen source were consistent with the putative role of the *PICST_54153* gene product as a β-alanine transaminase, since 1,3-diaminopropane and β-ureidopropionic acid are metabolic precursors of β-alanine (Fig. 1b). However, it was notable that growth on β-ureidopropionic acid failed altogether, while growth on 1,3-diaminopropane would only commence after a lag phase of at least 6 days. This was a cryptic observation considering that the catabolism of both β-ureidopropionic acid and 1,3-diaminopropane involve the extraction of one atom of nitrogen in the form of ammonia prior to the formation of β-alanine. Therefore, at least half of the available nitrogen from both β-ureidopropionic acid and 1,3-diaminopropane should be available to the yeast, which in turn would predict that the yeast would immediately display at least marginal growth even if it was unable to further metabolise β-alanine. The observed failure of the *∆picst_54153* mutant to grow on β-ureidopropionic acid and the severe lag phase observed on 1,3-diaminopropane, therefore, suggested that the accumulation of β-alanine during the catabolism of these two nitrogen sources might inhibit yeast growth in a *∆picst_54153* genetic background independently of nitrogen availability.

**Fig. 5:**
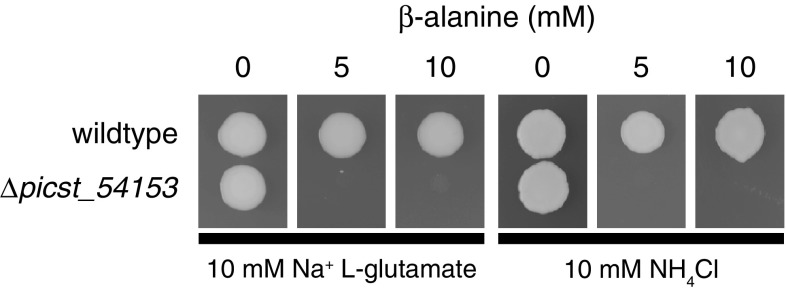
Growth inhibition on solid RSNLD medium (Linder 2018) of *Sch. stipitis* strains by β-alanine. Agarose (20 g/l) was used as a gelling agent instead of agar to avoid the introduction of additional nitrogen sources. *Sch. stipitis* strains TLSS001 (wildtype control) and TLSS011 (*∆picst_54153*) were diluted to OD_600_ 0.1 in sterile water and 2 μl of each cell suspension was deposited on the agarose plates. Plates were photographed after 6 days incubation at 30 °C

The inhibitory potential of β-alanine on *Sch. stipitis* growth was therefore tested using a simple solid media spot assay. Equal amounts of cells from the *∆picst_54153* mutant and the reference strain were deposited on RSNLD agarose plates containing a preferred nitrogen source (10 mM of either sodium l-glutamate or NH_4_Cl) and increasing amounts of β-alanine. (Agarose was used instead of agar as a gelling agent to prevent the introduction additional nitrogen compounds.) After a 6-day incubation, the *∆picst_54153* mutant and reference strain were indistinguishable on solid media containing either sodium l-glutamate or NH_4_Cl without β-alanine supplementation (Fig. 5). However, the *∆picst_54153* mutant failed to grow if the media was supplemented with at least 5 mM β-alanine irrespective of the presence of other nitrogen sources. This demonstrated a clear inhibitory effect of β-alanine on the *∆picst_54153* mutant independently of nitrogen availability.

## Discussion

The genetics of nitrogen assimilation in non-*Sac. cerevisiae* species of yeast (so-called “non-conventional yeasts”) remain poorly understood. It has long been known that the range of nitrogen sources that are assimilated by *Sac. cerevisiae* is relatively limited compared to other species of budding yeasts (Di Carlo et al. 1952; Large 1986; Linder 2014). The present study employed a reverse genetics approach to identify the product of the *Sch. stipitis* gene *PICST_54153* as a likely β-alanine transaminase. The phenotypical data reported here need to be followed up by biochemical studies of the purified Picst_54153 protein to establish its substrate specificity. Detectable impairment in growth on 1,4-diaminobutane and 1,5-diaminopentane (Fig. 4) would suggest that the Picst_54153 protein participates to some degree in the transamination of the predicted downstream intermediates GABA and δ-aminovaleric acid, respectively. Yet, no comparable growth defect was observed in the *∆picst_54153* mutant when GABA and δ-aminovaleric acid were provided as nitrogen sources. One possibility is that differences in intracellular localisation of the catabolic pathways for diamines and ω-amino acids could preclude the Picst_54153 protein from participating in the transamination of ω-amino acids imported directly from the cell exterior. Another possibility is that the *PICST_54153* gene is activated in response to the presence of the diamines 1,4-diaminobutane and 1,5-diaminopentane in the external environment, but not in response to GABA or δ-aminovaleric acid. The function and substrate specificity of the putative *Sch. stipitis* Uga1 GABA transaminase orthologue encoded by the *PICST_46781* gene also needs to be elucidated to better understand *Sch. stipitis* ω-amino acid assimilation. The question of assigning a common gene name to the *PICST_54153* gene in *Sch. stipitis* remains open. The name *PYD4* would reflect its necessity for the catabolism of β-ureidopropionic acid and the *Sch. stipitis* genome does contain a putative orthologue (systematic gene name *PICST_28429*) of the *L. kluyveri PYD3* gene. However, the phylogenetic analysis suggested independent duplication events of the ancestral *UGA1* gene to produce the *L. kluyveri PYD4* gene and the *Sch. stipitis PICST_54153* gene (Fig. 3). A more comprehensive phylogenetic and functional study of the ω-amino acid transaminase cluster across a representative selection of budding yeast species should be conducted before a standardised gene nomenclature can be adopted.

The inhibitory property of β-alanine on the *∆picst_54153* mutant (Fig. 5) was unexpected. The initial identification of the *PYD4* gene in *L. kluyveri* noted that the initial mutagenised strain (*pyd41*) was unable to use dihydrouracil or β-ureidopropionic acid as nitrogen source (Andersen et al. 2007), despite the initial extraction of one nitrogen atom in the form of ammonia through hydrolysis of the ureido group of β-ureidopropionic acid by the enzyme ureidopropionase (encoded by the *PYD3* gene). This result mirrors the observations in the present study that a non-functional β-alanine transaminase also precludes utilisation of nitrogen extracted at an earlier stage in the catabolic pathway. However, a complete deletion of the *PYD4* gene in *L. kluyveri* did allow for weak growth on β-alanine, while growth on dihydrouracil and β-ureidopropionic acid appeared unaffected (Andersen et al. 2007). The ability of the *pyd41* and *Δpyd4* mutants to utilise 1,3-diaminopropane as a nitrogen source was never reported by the authors of the study. The exact molecular nature of the *pyd41* mutation in *L. kluyveri* remains unknown at the time of writing. The phenotypes of the two different *PYD4* mutations in *L. kluyveri* indicate that there could exist a redundant β-alanine transaminase activity in this yeast, but that *PYD4* gene product somehow determines the inhibitory potential of β-alanine in a manner that may not involve its enzymatic activity. Growth inhibition by β-alanine has also been reported in *Sac. cerevisiae* (Cartwright et al. 2012). The inhibitory mechanism of β-alanine is unknown at present, as is the mechanism by which the *PICST_54153* gene prevents growth inhibition. One possibility is that the Picst_54153 transaminase metabolises β-alanine even in the presence of a preferred nitrogen source. β-alanine consumption in the presence of l-glutamic acid or NH_4_Cl was not assayed in the current study, but such an experiment would address whether the enzymatic activity of the Picst_54153 transaminase is directly involved in preventing growth inhibition by β-alanine.

The abundance of recently sequenced genomes from non-conventional yeasts has resulted in a growing list of so-called “orphan genes” meaning predicted genes lacking experimentally verified functions (Hanson et al. 2009). Concurrent with the initial identification of the *PICST_54153* gene as a potential ω-amino acid transaminase in the present study, several other putative class III transaminases were identified in the genomes of *L. kluyveri* and *Sch. stipitis* that did not cluster with previously characterised yeast enzymes (Fig. 3). The *L. kluyveri SAKL0H16522g* gene was of particular interest as it did not cluster with any other class III transaminase in *Sac. cerevisiae* or *Sch. stipitis*. A sequence similarity search against the SwissProt database and Protein Data Bank identified a bifunctional 2,2-dialkylglycine decarboxylase/transaminase from the betaproteobacterium *Burkholderia cepacia* (GenBank protein accession AAA50844; Keller et al. 1990), which was 54% identical and 73% similar to the predicted *L. kluyveri* gene product. The biological function of this enzyme is presently unclear. A previously published report of the homologous gene in the wheat blotch fungus *Mycosphaerella graminicola* (GenBank protein accession AAM18795) found that the deletion of the gene did not affect morphology or virulence (Adachi et al. 2003). A BLASTP search of the *L. kluyveri* Sakl0h16522p protein sequence against the protein complement of other budding yeast genomes in GenBank identified likely orthologues in a number of species, although the taxonomic distribution was notably scattered across several families (data not shown). A separate cluster consisting of four uncharacterised class III transaminases from *L. kluyveri* and *Sch. stipitis* (Fig. 3) did not return any likely orthologues from the SwissProt database and Protein Data Bank. The functions of these four orphan genes (*PICST_35479, PICST_38281, SAKL0E05016g, SAKL0F16566g*) remain to be determined.

## Abbreviations

GABA: γ-Amino butyric acid
IGR: Intergenic region
RSNLD: Reduced sulphur/nitrogen-limited glucose medium
